# Interruptible demyelination in avian riboflavin deficient neuropathy

**DOI:** 10.1186/s13578-024-01233-5

**Published:** 2024-04-22

**Authors:** Zhao Cai

**Affiliations:** 1 Division of Anatomical Pathology, SA Pathology, Royal Adelaide Hospital, Adelaide, SA 5000 Australia; 2 School of Medicine, Faculty of Health & Medical Science, University of Adelaide, Adelaide, Australia

**Keywords:** Riboflavin, Demyelination, Remyelination, Teased nerve fibre, Schwann cell

## Abstract

**Background and aims:**

The evolution of demyelination in individual internodes remains unclear although it has been noticed the paranodal demyelination precedes internodal demyelination in neuropathies with diverse aetiologies. For therapeutic purpose, it is fundamental to know whether the demyelinating procedure in affected internodes can be interrupted. This study aimed to delineate the development of demyelination in individual internodes in avian riboflavin deficient neuropathy.

**Methods:**

Newborn broiler meat chickens were maintained either on a routine diet containing 5.0 mg/kg riboflavin, a riboflavin deficient diet containing 1.8 mg/kg riboflavin, or initially a riboflavin deficient diet for 11 days and then routine diet plus riboflavin repletion from day 12. Evolution of demyelination in individual internodes was analyzed by teased nerve fibre studies from day 11 to 21.

**Results:**

In riboflavin deficient chickens, demyelination was the predominant feature: it was mainly confined to the paranodal region at day 11; extended into internodal region, but less than half of the internodal length in most affected internodes at day 16; involved more than half or whole internode at day 21. In the internode undergoing demyelination, myelin degeneration of varying degrees was noticed in the cytoplasm of the Schwann cell wrapping the internode. Two days after riboflavin repletion, co-existence of remyelination and active demyelination within individual internodes was noticed. Remyelination together with preserved short original internodes was the characteristic feature 4 and 9 days after riboflavin repletion.

**Conclusion:**

Riboflavin repletion interrupts the progression from paranodal to internodal demyelination in riboflavin deficient chickens and promotes remyelination before complete internodal demyelination.

## Introduction

Segmental demyelination, firstly described in lead neuropathy in guinea pig by Gombault in 1880, refers to myelin degeneration of a paranode (paranodal segmental demyelination) or an internode (internodal segmental demyelination) [1]. Early changes at the paranodal region have been found in demyelination of many types either primary, such as inflammatory [2, 3] and toxic [4–6] or secondary to axonal change [7]. It is believed that paranodal demyelination precedes internodal demyelination in both the peripheral nervous system (PNS) [8] and central nervous system (CNS) [9, 10]. However, the procedure from paranodal to complete internodal demyelination has not been previously described. For potential therapeutic purpose, it is fundamental to know whether the progression from paranodal to internodal demyelination is an irreversible process once initiated or whether the demyelinating procedure in affected internodes can be interrupted.

Riboflavin (a water-soluble vitamin, vitamin B2, VB_2_) and its derivatives are coenzymes for numerous oxidases and dehydrogenases in eukaryotic cells [11]. Riboflavin deficiency leads to impaired β-oxidation of fatty acids [12] and significant change in proteomic profiles [13]. New-born chickens fed with riboflavin-deficient (VB_2_^−^) diet develop a demyelinating peripheral neuropathy [6, 14–17]. Remyelination and spontaneous recovery after about 3 weeks may be due to endogenous microbial synthesis of riboflavin in the intestine (absent in newborn chicks) [18]. Previous studies in avian riboflavin deficient neuropathy found early changes at the paranodal region [15, 16], implying beginning of this pathogenic process. Here, by way of teased nerve fibre (TF) studies, we delineated the evolution of demyelination in affected internodes with the focus on interruption of this demyelinating procedure by VB_2_ repletion (VB2^+^).

## Materials and methods

The avian riboflavin deficiency model of demyelinating peripheral neuropathy has previously been described [15]. In the current study, newborn broiler meat chickens (Cobb 500, Cobb-Vantress Inc, Arkansas, USA) were maintained ad libitum on (1) a conventional diet containing 5.0 mg/kg riboflavin, control groups; (2) a deficient diet containing 1.8 mg/kg riboflavin, VB_2_^−^ groups; (3) initial riboflavin deficient diet for 11 days and then from post hatch day 12 (PH12d) conventional diet plus intraperitoneal injection of 0.5 ml Vitamin B Complex Injection containing 0.5 mg/ml VB2 (Ceva Animal Health, Australia), VB_2_^−/+^ groups. Five animals in each group were studied. Control and VB_2_^−^ chickens were killed on PH11d, PH16d and PH21d, and VB_2_^−/+^ chickens on PH14d, PH16d and PH21d by transcardiac perfusion with 4% paraformaldehyde 0.1 M phosphate buffer (pH 7.4). Left sciatic and brachial nerves were processed according to a standard peripheral nerve biopsy protocol for light microscopy (LM) and electron microscopy (EM) [19]. Right sciatic and brachial nerves were prepared for sectional studies of resin teased nerve fibres (TFs) according to a published method [20]. In brief, after the perfusion, right sciatic and brachial nerves were further fixed in 4% paraformaldehyde/2.5% glutaraldehyde in 0.1 M phosphate buffer for 1.5 h and postfixed in 1% osmium tetroxide for 2 h. After washing in the cacodylate buffer, nerves were dehydrated in graded ethanol, softened in fresh epoxy resin for 3 days. Individual nerve fibres were isolated in fresh epoxy resin and mounted onto a carbon-coated slide. Fifty TFs from each nerve were prepared. Some fibres were further marked at specific sites using short TFs as “marker” fibres and left in an oven for resin polymerization. A small capsule (for routine electron microscopy resin embedding) filled with fresh resin was placed upside down onto the carbon-slide, covering the area that contained the teased fibres, and left in the oven for resin polymerization. The under surface of the slide was placed on top of a solid metal block, which was pre-cooled in liquid nitrogen. Due to the temperature difference, the resin block separated from the slide along the line of the carbon coat, with the fibres remaining on the surface of the resin block. Longitudinal sections of individual nerve fibres were collected from this resin block using an ultramicrotome. This resin block may also be trimmed around the fibres and placed in a capsule filled with fresh resin, keeping the teased fibres parallel to the long axis of the capsule, and the resin polymerized as above. The fibres were then transversely sectioned at specified sites to correlate with abnormalities seen on teased preparation.

## Results

As reported previously, no neurological signs and pathological changes were found in control animals; VB_2_^−^ chickens showed progressive weakness and paresis from PH8d until end of this study [15]. The paresis in VB_2_^−/+^ chickens became stable at PH16d (4 days after VB_2_ repletion), and gradually recovered thereafter. Routine pathological examination (Table 1) revealed predominant demyelination in all VB_2_^−^ groups and predominant remyelination in VB_2_^−/+^ chickens at PH16d and PH21d. Endoneurial oedema, hypertrophic Schwann cells with lipid deposition, complex myelin foldings with varying stages of degeneration and myelin fragments in Schwann cell cytoplasm, as noticed in VB_2_^−^ chickens [15], were also found in VB_2_^−/+^ chickens at less severity. But there were no obvious difference of hypertrophic fibroblasts and onion bulb formation [14] between VB_2_^−^ and VB_2_^−/+^ chickens at the same time points. Macrophage-mediated myelin stripping, as described in both human and animal inflammatory demyelinating neuropathies [3, 21], was not found here. Sciatic and brachial nerves showed same pathological changes.

**Table 1:** Plastic section study of the peripheral nerves^a^

Group	EE	Dem	Rem	AD	MF	HSC	LD	FOB
Control PH11d	−	−	−	−	−	−	−	−
Control PH16d	−	−	−	−	−	−	−	−
Control PH21d	−	−	−	−	−	−	−	−
VB_2_^−^ PH11d	+	+	−	−	+	+	+	+
VB_2_^−^ PH16d	+++	++	±	±	+++	+++	+++	++
VB_2_^−^ PH21d	+++	+++	+	+	++	+++	+++	+++
VB_2_^−/+^ PH14d	++	+	++	±	++	+++	++	++
VB_2_^−/+^ PH16d	+	+	+++	−	+	++	+	++
VB_2_^−/+^ PH21d	+	±	+++	−	±	++	+	+++

^a^Refer to [14, 15] for detailed description

*EE* endoneurial edema, *Dem* demyelination, *Rem* remyelination, *AD* axonal degeneration, *MF* myelin foldings, *HSC* hypertrophic Schwann cell, *LD* lipid deposit, *FOB* fibroblast onion bulb

### TF studies in VB_2_^−^ chickens

**Fig. 1:**
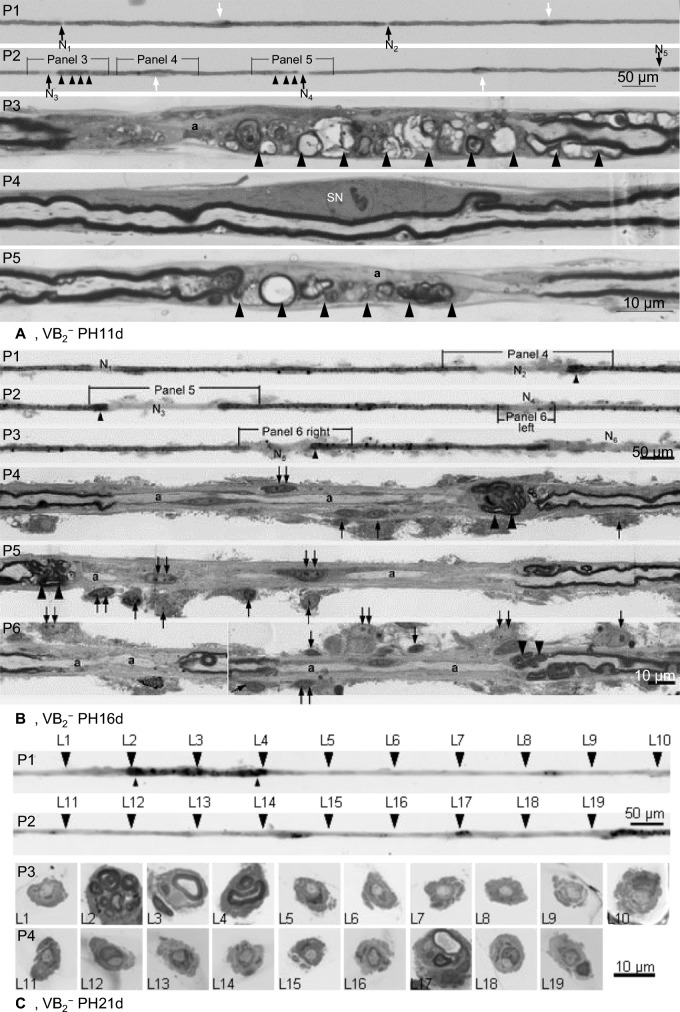
Evolution of demyelination in VB_2_^−^ chickens. Light micrographs. **A** Panel 1 (P1) and P2 show part of a TF from a VB_2_^−^PH11d chicken. The nodes of Ranvier are indicated by arrows (N_1_ to N_5_). Paranodal demyelination is noted at 2 sites (N_3_ and N_4_) and normal node of Ranvier at 3 sites (N_1_, N_2_ and N_5_). Schwann cell nuclei (white arrowheads) are located in the middle of each internode. P3 to P5 are longitudinal sections through designated segments. P3 is through the first paranodal demyelinating area. No discernible myelin sheath is seen in the demyelinated segment. Myelin degeneration (large arrowheads in P3) is seen in the internodal region adjoining the paranodal demyelination (small arrowheads in P2). P4 is through the central part of the internode with paranodal demyelination. The myelin sheath is well preserved in this segment and a Schwann cell nucleus (SN) is present. P5 is through the second paranodal demyelinating area. No discernible myelin sheath is seen in the demyelinated segment. Myelin degeneration (large arrowheads in P5) is seen in longitudinal section through the segment (small arrowheads in P2) adjoining the paranodal demyelination. The axon is attenuated in demyelinating regions. **B** (adapted from Cai et al., 2007 [14] with permission from the author and publisher): P1 to P3 show a TF from a VB_2_^−^PH16d chicken displaying paranodal demyelination (N_1_ and N_4_) and partial internodal demyelination (N_2_, and N_3_, N_5_ and N_6_). Paranodal swellings (small arrowheads) and surrounding pale-osmicated material representing the fibroblastic onion bulb like proliferation [14]. P4 to P6 are longitudinal sections through designated segments in the three upper panels. In the demyelinated segments, there is no discernible myelin sheath and axon (a) is attenuated. Redundant myelin foldings with varying degrees of myelin splitting and degeneration (large arrowheads) are found in longitudinal sections through paranodal swellings while the myelin sheaths in the neighbouring non-demyelinated internodal regions are intact. The fibre is surrounded by a variably thick fibroblastic proliferation consisting of fibroblast processes and collagen fibres. Numerous fibroblast nuclei (arrows), some with multiple distinct nucleoli (double arrows), are noted at both demyelinated and non-demyelinated segments. **C** P1 and P2 show part of a TF from a VB_2_^−^PH21d chicken displaying internodal demyelination. P3 and P4 are cross sections through designated sites of the TF, showing denuded axon in the demyelinated segments (L1, 5–19), redundant myelin foldings with myelin splitting and degeneration at both ends of the myelin-maintained segment (L2 and 4). The axon size in the region with intact myelin (L3) is larger than that of demyelinated regions. Fibroblast processes and collagen ensheath the TF at all levels to some degree

Fifty TFs were prepared from each animal. In control animals, at least 5 consecutive internodes in each fibre were examined. Pathological nerve fibres with similar length were examined. Demyelination was the predominant change in TF preparations from all VB_2_^−^ groups. At PH11d (Fig. 1A), demyelination was mainly confined to the paranodal region, covering not more than 10 percent internodal length, paranodal demyelination. Normal internodes and demyelination were often noticed in the same fibres. At PH16d (Fig. 1B), paranodal demyelination was present in some internodes. In majority of affected internodes, demyelination involved both paranodal and adjoining internodal region, covering more than 10 percent of internodal length and the demyelinated segment not longer than that of neighbouring myelin sheath retained segment, partial internodal demyelination. In some affected internodes, demyelination involved whole internode or the demyelinated segment longer than that of neighbouring myelin retained segment, internodal demyelination. Demyelination of different types were often noticed in the same fibres. At PH21d (Fig. 1C), internodal demyelination was the major type. Quantitative analyses (Table 2) confirmed: the average percentage of paranodal demyelination decreased significantly with age, whereas the percentage of internodal demyelination increased significantly with age; the percentage of partial internodal demyelination increased significantly from P11d to P16d and then decreased significantly at P21d. These results suggest the evolution of demyelination in VB_2_^−^ chickens: starting from paranodal region, progressing into internodal region, and finally involving whole internode.

**Table 2:** Frequency of different types of demyelination in VB_2_^−^ chickens

	Paranodal demyelination	Partial internodal demyelination	Internodal demyelination
PH11d	84 ± 5.48	16 ± 5.48	0
PH16d	19 ± 2.65**	62.8 ± 4.66*	18.2 ± 5.22
PH21d	5.6 ± 1.51**	15.4 ± 3.91**	79 ± 3.54*

ANOVA and student *t* tests revealed significantly higher (*) or lower (**) than that at the previous time point (P < 0.05)

Sectional studies were performed in 50 TFs with different types of demyelination (Fig. 1). “Naked” axon, axon without surrounding myelin sheath, was present in the demyelinated regions of various types. It was better demonstrated in longitudinal sections of TFs (Fig. 1A and B): active myelin degeneration in myelin retained segments, more severe close to the denuded site; and well-preserved myelin and Schwann cell nucleus in the middle. Myelin fragments was often noticed in the cytoplasm of the Schwann cell enwrapping the internode, which was better demonstrated in transverse sections of TFs (Fig. 1C). Macrophage stripping myelin, as reported in inflammatory demyelinating neuropathies, was not found here by electron microscopy in both transverse and longitudinal sections of TFs. The axon in the demyelinated sites were preserved but often attenuated. These findings further confirmed the evolution of demyelination, proceeding from paranodal gradually into internodal region, and suggest the “myelinating” Schwann cells play a pivotal in myelin degeneration and clearance.

Like previously reported, enveloping fibroblastic reaction [14] was noticed in all groups, but more prominent at PH16d and PH21d.

### TF studies in VB_2_^−/+^ chickens

**Fig. 2:**
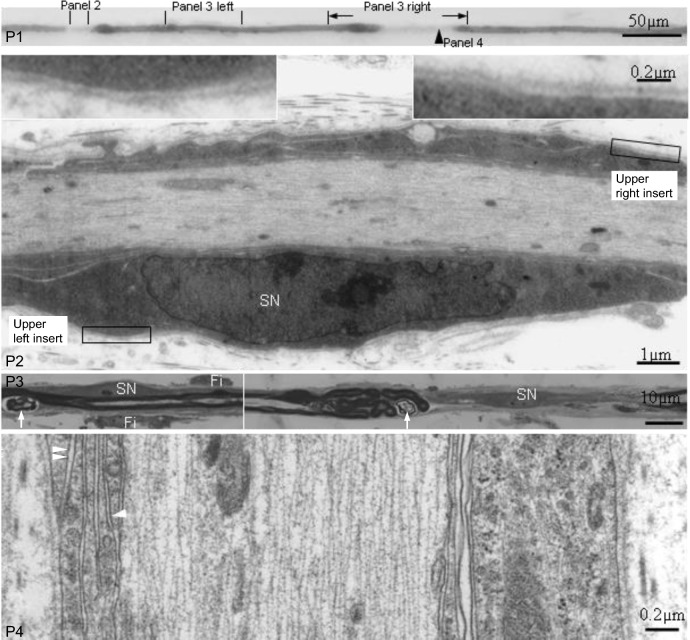
Light micrographs, P1 and P3; Electron micrographs, P2 and P4. P1 is part of a TF from a VB_2_^−/+^PH14d chicken displaying paranodal and partial internodal demyelination according to the surface appearance. P2 is a longitudinal section through the paranodal demyelination segment. A Schwann cell nucleus (SN) is present. This Schwann cell envelops the axon under basal lamella of the nerve fibre (inserts). P3 left is a longitudinal section through the middle of the neighbouring myelin-maintained segment showing a Schwann cell nucleus (SN), 2 attached fibroblasts (Fi) and myelin debris (white arrows). P3 right is a longitudinal section through the partial internodal demyelination segment. A centrally located Schwann cell nucleus (SN) is present. Redundant myelin foldings with myelin breakdown (white arrows) is present in the paranodal region of the neighbouring myelin-maintained segment. P4 is a longitudinal section (rotate 90°) through the partial internodal demyelination region (arrowhead in panel 1) showing 4 layers of non-compacted myelin lamellae surrounding the axon under the basal lamella of the nerve fibre: an inner mesaxon (white arrowhead) connecting the axolemma and outer mesaxon (double white arrowheads) connecting Schwann cell basement membrane

**Fig. 3:**
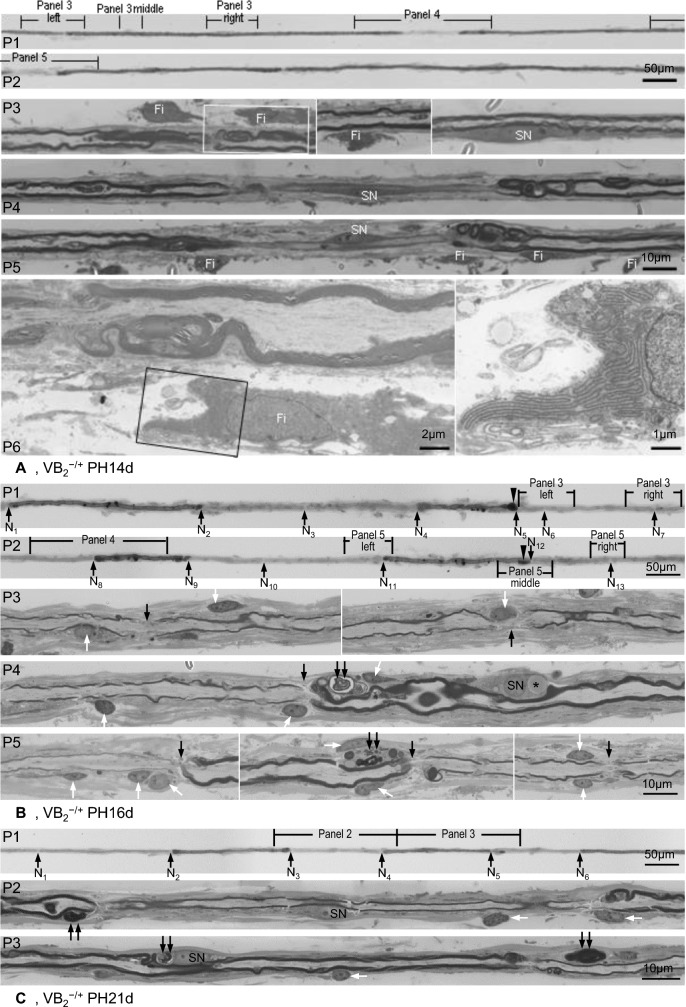
Evolution of demyelination and remyelination in VB_2_^−/+^ chickens. Light micrographs except for electron micrograph at P6 in **A**. **A** P1 and P2 are part of a TF from a VB_2_^−/+^PH14d chicken with partial internodal demyelination. P3 to P5 are longitudinal sections through designated sites of the TF. Redundant myelin foldings are seen in externally normal paranodal regions (P3 left) and paranodal regions adjoining partial internodal demyelination (P4 and P5). A centrally located Schwann cell nucleus (SN) is noticed in the demyelinated regions (P4 and P5) and neighbouring myelin-maintained segment (P3 right). Supernumerary fibroblasts (Fi) are seen attached to the TF preparation at paranodal and internodal regions. Electron microscopy (P6) further demonstrates the fibroblast with enriched rough reticulum (white rectangle in P3 left). **B** P1 and P2 are part of a TF from a VB_2_^−/+^PH16d chicken, showing remyelination. P3 to P5 are longitudinal sections of designated segments. The nodes of Ranvier (N_1_–N_13_ arrows) are hardly seen from the surface appearance of the TF, but are clearly identified in longitudinal sections. The 1st (N_1_–N_2_), 4th (N_4_–N_5_), 8th (N_8_–N_9_) and 11th (N_11_–N_12_) internodes are original internodes with thick myelin sheaths. The 2nd (N_2_–N_3_), 3rd (N_3_–N_4_), 5th (N_5_–N_6_), 6th (N_6_–N_7_), 7th (N_7_–N_8_), 9th (N_9_–N_10_), 10th (N_10_–N_11_) and 12th (N_12_–N_13_) internodes are remyelinating internodes with thin myelin sheaths. Substantial variation of the internodal length is seen in both original and remyelinating internodes. The length of some remyelinating internodes, such as the 2nd (N_2_–N_3_), 3rd (N_3_–N_4_), 7th (N_7_–N_8_) and 10th (N_10_–N_11_) internodes, are similar or even longer than some original internodes, such as the 4th (N_4_–N_5_) and 8th (N_8_–N_9_) internodes. Focal myelin swellings (arrowheads) are present at the paranodal regions of original internodes. The nodal gap is not extra ordinarily large, implicating that the length of the remyelinating internode is already fixed at this stage. A Schwann cell nucleus (SN) and lipid deposition (asterisk) are present in the middle of an original internode. Myelin debris is seen in the paranodal Schwann cell cytoplasm of the original internode (double arrows). The TF is surrounded by a variably thick fibroblastic proliferation consisting of fibroblast processes and collagen fibres. Numerous fibroblast nuclei (white arrows) are noted in the longitudinal section at both original and remyelinating internodes. **C** P1 is part of a TF from a VB_2_^−/+^PH21d chicken, showing remyelination. The nodes of Ranvier are indicated by arrows (N_1_–N_6_). There is no considerable variation of the internodal length between the original (N_2_–N_3_ and N_4_–N_5_) and remyelinating internodes (N_1_–N_2_, N_3_–N_4_ and N_5_–N_6_). One of the remyelinating internode (N_1_–N_2_, 194 µm in length) is even longer than that of original internodes (N_2_–N_3_, 176 µm; N_4_–N_5_, 159 µm). P2 and P3 are longitudinal sections through the full length of a remyelinating and a neighbouring original internode in the first panel. Schwann cell nucleus (SN) is present in the middle of both the remyelinating and original internodes. Degenerating myelin of varying stages (double arrows) is noticed in Schwann cell cytoplasm of both original and remyelinating internodes. White arrows indicate fibroblast nuclei

At PH14d (2 days after riboflavin repletion), paranodal and partial internodal demyelination were the predominant change according to the surface appearances (Figs. 2 and 3A). But sectional studies revealed the following features in 14/20 examined such “demyelinating” fibres: (1) a Schwann cell nucleus in the “denuded” region, even a very short segment (50 µm), e.g., panels 2 and 3 right in Fig. 2 and panels 4 and 5 in Fig. 3A; (2) a few numbers of myelin lamellae around the axon with inner mesaxon connecting the axon and outer mesaxon connecting the Schwann cell basal membrane, indicating this Schwann cell was myelinating the axon, e.g., panel 4 in Fig. 2; (3) active myelin degeneration at the peripheries of myelin retained segments (here named original internodes), e.g. panel 3 right in Fig. 2 and panels 4 and 5 in panel 3A. These results are interpreted as co-existence of remyelination and active demyelination within affected internodes.

At PH16d and PH21d (4 and 9 days after riboflavin repletion), remyelination (thin myelin sheath) was present in majority of TFs. In these remyelinating fibres, the characteristic feature was the short original internodes (with thick myelin sheaths), especially at PH21d: they were usually irregular, the shortest less than half of the longest and even some remyelinating internodes (Fig. 3B); sometimes regular, similar to the length of adjacent remyelinating internodes (Fig. 3C). Sectional studies of 38 such fibres revealed: (1) new nodes of Ranvier, which were hardly visible from the surface appearance especially at PH16d (Fig. 3B); (2) active myelin degeneration at the peripheries of original internodes at PH16d (Fig. 3B), but much less in frequency and severity at PH21d (Fig. 3C); (3) myelin debris in the cytoplasm of myelinating Schwann cell encompassing the original (e.g., panels 4 and 5 middle in Fig. 3B and panel 3 in Fig. 3C) and sometimes remyelinating internodes (e.g., panel 3 in Fig. 3C). These findings are interpreted as: the progression of demyelination within individual internodes was interrupted, at least alleviated; both the original myelinating Schwann cell and remyelinating Schwann cell involve in myelin clearance.

Enveloping fibroblasts as seen in VB2^−^ animals were also present in VB2^−/+^ animals (Figs. 2 and 3).

## Discussion

### Demyelination in VB2^−^ chickens proceeds from paranodal to central internodal region and is driven by Schwann cell

In the current study, morphometric analyses revealed significant changes from predominant paranodal demyelination at PH11d, to predominant partial internodal demyelination at PH16d, and then predominant internodal demyelination at PH21d. Sectional studies of TFs further demonstrated incipient myelin degeneration at paranodal and later at internodal region. These findings confirmed that, as expected, demyelination in VB_2_^−^ chickens proceeds from paranodal to internodal region until the full length of the internode is affected.

It is generally accepted that macrophage-mediated myelin degeneration and clearance play the pivotal role in the pathogenic mechanism in demyelinating neuropathies with various aetiologies. However, the concept of macrophage-mediated demyelination ignores morphological facts: myelin debris is more commonly found in Schwann cells than macrophages. Macrophage-mediated myelin stripping was not found in the current and previous studies by electron microscopy and immunohistochemistry. In contrast, varying stages of degenerated myelin fragments were found in the cytoplasm of hypertrophic Schwann cells [14–17], consistent with the myelin phagocytosis and clearance function of Schwann cell [22, 23]. Sectional studies of TFs here proved that the Schwann cell expressing such myelinolytic activity was the same Schwann cell enwrapping the internode, indicating that the original myelinating Schwann cell plays a critical role in initiating and promoting demyelination within the same internode. Such Schwann cell is named demyelinating Schwann cell [24]. The longitudinal extension of myelin degeneration and clearance from paranodal to internodal region by demyelinating Schwann cells is named Demyelination Driven by Demyelinating Schwann Cell (DDDSC), which is thought the common pathway of demyelination in immune-independent and some inflammatory demyelinating neuropathies [24, 25].

### Interruptible demyelination and remyelination in VB2^−/+^ chickens

Paranodal origin of demyelination has been recognized in neuropathies with diverse aetiologies in both PNS and CNS [8–10]. It is commonly accepted: two or more Schwann cells remyelinate one completely demyelinated internode, resulting in short remyelinated internodes. It remains unclear: (1) whether remyelination could occur before one internode is completely demyelinated? (2) whether the demyelinating process within affected internodes could be stopped? Although there has been no previous study specifying the possibility to interrupt the evolution from paranodal to internodal demyelination, it is believed that whenever initiated, DDDSC will progress unidirectionally toward complete removal of intracellular myelin sheath debris before remyelination happens. This process is so-called irreversible demyelination [24]. But in the notion of irreversible demyelination [24], two facts are overlooked, rapid progression of the disease and continuous, even irreversible, effect of the pathogen. As the progression from paranodal to complete internodal demyelination takes a few (5–10) days in the current avian model, it provides a time window to interfere with this procedure. Importantly, the pathogenic factor, VB_2_^−^, is readily rectified by VB_2_^+^. VB_2_^+^ was applied from PH12d as myelin degeneration in most affected internodes was limited to the paranodal and adjoining regions with preservation of central internodal myelin segments at this time. Co-existence of remyelination and active demyelination within individual internodes in VB_2_^−/+^PH14d chickens indicates occurrence of remyelination before complete internodal demyelination. Presence of short original internodes in remyelinating fibres, especially at PH21d, suggests the demyelinating procedure in affected internodes be interrupted. This type of demyelination and remyelination in avian riboflavin deficiency is here named as interruptible demyelination driven by Schwann cell (IDDSC). Whether IDDSC applies to avian riboflavin deficiency only? The morphological features, myelin debris in Schwann cell cytoplasm, intercalated internode and so called “hypomyelination” noticed by conventional pathological examinations [1, 26, 27], suggest that IDDSC apply to demyelinating neuropathies with diverse aetiologies. In clinical practice, symptoms in some patients with demyelinating neuropathies improve soon after treatment. It may attribute to the interruptible process of demyelination. Studies in human samples, especially sectional studies of TFs, will test this speculation.

The current study provides morphological evidence. Molecular mechanisms for interruptible demyelination remain further studies. Because of the specific feature of myelinated fibres, long and segmental, it is proposed that investigation of dynamic molecular expressions, including related Schwann cell phenotypes, along a decent length of same fibres, such as multiple consecutive internodes, will be an important next step. It is anticipated that novel therapies to demyelinating neuropathies with diverse aetiologies may be developed accordingly.

In conclusion, demyelination in avian VB_2_^−^ neuropathy proceeds from paranodal gradually to internode region; the Schwann cell, originally myelinating the internode, plays a pivotal role in this demyelinating procedure, myelin degeneration and clearance; VB_2_^+^ interrupts the demyelinating procedure in affected internodes and promotes remyelination before complete internodal demyelination.

## Data Availability

The data and materials are available.

## References

[1] Dyck PJ, Dyck PJB, Engelstad J, Dyck PJ, Thomas PK, Griffin J (2005). Pathologic alterations of nerve. Peripheral neuropathy.

[2] Koike H, Kadoya M, Kaida KI (2017). Paranodal dissection in chronic inflammatory demyelinating polyneuropathy with anti-neurofascin-155 and anti-contactin-1 antibodies. J Neurol Neurosurg Psychiatry.

[3] Koike H, Katsuno M (2021). Macrophages and autoantibodies in demyelinating diseases. Cells.

[4] Allt G, Cavanagh JB (1969). Ultrastructural changes in the region of the node of Ranvier in the rat caused by diphtheria toxin. Brain.

[5] Uncini A, England JD, Rhee EK (1988). Tellurium-induced demyelination: an electrophysiological and morphological study. Muscle Nerve.

[6] Weller RO, Nester B (1972). Early changes at the node of Ranvier in segmental demyelination. Histochemical and electron microscopic observations. Brain.

[7] Dyck PJ, Lais AC, Karnes JL (1981). Permanent axotomy, a model of axonal atrophy and secondary segmental demyelination and remyelination. Ann Neurol.

[8] Bilbao JM, Schmidt RE (2015). Biopsy Diagnosis of Peripheral Neuropathy.

[9] Fu Y, Frederick TJ, Huff TB (2011). Paranodal myelin retraction in relapsing experimental autoimmune encephalomyelitis visualized by coherent anti-Stokes Raman scattering microscopy. J Biomed Opt.

[10] Stojic A, Bojcevski J, Williams SK, Diem R, Fairless R (2018). Early nodal and paranodal disruption in autoimmune optic neuritis. J Neuropathol Exp Neurol.

[11] Rivlin RS, Bowman BA, Russell RM (2001). Riboflavin. Present knowledge in nutrition.

[12] Ross NS, Hansen TP (1992). Riboflavin deficiency is associated with selective preservation of critical flavoenzyme-dependent metabolic pathways. BioFactors.

[13] Xin Z, Pu L, Gao W, Wang Y, Wei J, Shi T, Yao Z, Guo C (2017). Riboflavin deficiency induces a significant change in proteomic profiles in HepG2 cells. Sci Rep.

[14] Cai Z, Blumbergs PC, Finnie JW (2007). Novel fibroblastic onion bulbs in a demyelinating avian peripheral neuropathy produced by riboflavin deficiency. Acta Neuropathol.

[15] Cai Z, Finnie JW, Blumbergs PC (2006). Early paranodal myelin swellings (tomacula) in an avian riboflavin deficiency model of demyelinating neuropathy. Exp Neurol Exp Neurol.

[16] Johnson WD, Storts RW (1988). Peripheral neuropathy associated with dietary riboflavin deficiency in the chicken. I Light microscopic study. Vet Pathol.

[17] Jortner BS, Cherry J, Lidsky TI (1987). Peripheral neuropathy of dietary riboflavin deficiency in chickens. J Neuropathol Exp Neurol.

[18] Summers BA, Cummings JF, de Lahunta A (1995). Veterinary neuropathology.

[19] Cash K, Blumbergs PC, Woods AE, Ellis RC (1995). Neuromuscular tissue. Laboratory histopathology: a complete reference.

[20] Cai Z, Cash K, Swift J (2001). A novel method for correlating internal and external structure of individual myelinated nerve fibres. J Neurosci Methods.

[21] Bensfield AC, Evans J, Pesayco JP (2011). Recurrent demyelination and remyelination in 37 young Bengal cats with polyneuropathy. J Vet Intern Med.

[22] Balakrishnan A, Belfiore L, Chu TH (2021). Insights into the role and potential of Schwann cells for peripheral nerve repair from studies of development and injury. Front Mol Neurosci.

[23] Jessen KR, Mirsky R (2019). The success and failure of the Schwann cell response to nerve injury. Front Cell Neurosci.

[24] Park HT, Kim JK, Tricaud N (2019). The conceptual introduction of the “demyelinating Schwann cell” in peripheral demyelinating neuropathies. Glia.

[25] Park HT, Kim YH, Lee KE (2020). Behind the pathology of macrophage-associated demyelination in inflammatory neuropathies: demyelinating Schwann cells. Cell Mol Life Sci.

[26] Kalichman MW, Chalk CH, Mizisin AP (1999). Classification of teased nerve fibers for multicenter clinical trials. J Peripher Nerv Syst.

[27] Sima AAF, Blaivas M, Garcia JH, Budka H, McKeever PE, Sarnat HB, Sima AAF (1997). Peripheral neuropathies. Neuropathology: the diagnostic approach.

